# Development and validation of the Alimetry Gut-Brain Wellbeing Survey: a novel patient-reported mental health scale for patients with chronic gastroduodenal symptoms

**DOI:** 10.3389/fpsyg.2024.1389671

**Published:** 2024-07-08

**Authors:** Mikaela Law, Isabella Pickering, Gayl Humphrey, Gabrielle Sebaratnam, Gabriel Schamberg, Katie Simpson, Chris Varghese, Peng Du, Charlotte Daker, I-Hsuan Huang, Sahib S. Khalsa, Armen Gharibans, Greg O'Grady, Christopher N. Andrews, Stefan Calder

**Affiliations:** ^1^Department of Surgery, The University of Auckland, Auckland, New Zealand; ^2^Alimetry Ltd., Auckland, New Zealand; ^3^Department of Psychological Medicine, The University of Auckland, Auckland, New Zealand; ^4^Department of General Surgery, Auckland City Hospital, Auckland, New Zealand; ^5^Auckland Bioengineering Institute, The University of Auckland, Auckland, New Zealand; ^6^Department of Gastroenterology, Te Whatu Ora - Waitematā, Auckland, New Zealand; ^7^Translational Research Center for Gastrointestinal Disorders (TARGID), University of Leuven, Leuven, Belgium; ^8^Laureate Institute for Brain Research, Tulsa, OK, United States; ^9^Oxley College of Health Sciences, University of Tulsa, Tulsa, OK, United States; ^10^Perelman School of Medicine, University of Pennsylvania, Philadelphia, PA, United States; ^11^The Insides Company, Auckland, New Zealand; ^12^The Division of Gastroenterology, Cumming School of Medicine, University of Calgary, Calgary, AB, Canada

**Keywords:** anxiety, depression, disorders of gut-brain interaction, psychometrics, mental health

## Abstract

**Objective:**

There is currently a lack of validated questionnaires designed specifically to assess mental health within patients with chronic gastroduodenal symptoms. This research describes the multi-phase process used to develop and validate a novel mental health scale for patients with chronic gastroduodenal symptoms, the Alimetry® Gut-Brain Wellbeing (AGBW) Survey.

**Methods:**

A patient-centered multi-phase process was implemented. In Phase 1, the most relevant concepts for this patient population were selected from existing mental health scales, using data from 79 patients. In Phase 2, an interdisciplinary panel of experts generated scale items. In Phase 3, the scale underwent pre-testing with gastroenterologists (*n* = 9), health psychologists (*n* = 3), and patients (*n* = 12), with feedback incorporated over multiple rounds. Lastly, the psychometric properties of the scale were assessed in a sample of 311 patients via an online survey.

**Results:**

The AGBW Survey comprises a patient preface, 10 close-ended questions, and an optional open-ended question. This multidimensional scale assesses general mental health, alongside specific subscales relating to depression, stress, and anxiety. The subscale and total scores demonstrated high internal consistency (*α* = 0.91 for the total scale; *α* = 0.72–0.86 for subscales) and good convergent, divergent, concurrent validity, and known groups validity, with large effect sizes.

**Conclusion:**

The AGBW Survey is a brief, valid, and reliable scale for assessing mental health in patients with chronic gastroduodenal symptoms. It can be used as a tool to complement physiological tests and has the potential to guide psychological referrals, inform multidisciplinary management, and evaluate treatment outcomes.

## Introduction

Chronic gastroduodenal symptoms, like those experienced in gastroduodenal disorders of gut-brain interaction (DGBIs), affect more than 10% of the global adult population and constitute a significant health issue due to their increasing prevalence and rising healthcare costs (Drossman, 2016; Stanghellini et al., 2016; Schmulson and Drossman, 2017; Sperber et al., 2021). These symptoms include chronic nausea, vomiting, belching, regurgitation, epigastric pain/burning, early satiation, and excessive fullness, which are often experienced in conditions such as functional dyspepsia, gastroparesis, and chronic nausea and vomiting syndrome (Drossman, 2016; Stanghellini et al., 2016; Schmulson and Drossman, 2017). The management and diagnosis of these symptoms present considerable challenges as such patients frequently exhibit overlapping symptomology devoid of identifiable structural aetiology (Pasricha et al., 2021; Lacy et al., 2022). Consequently, there are limited well-defined diagnostic pathways and targeted treatment approaches for these patients (Stanghellini et al., 2016; Raubinger et al., 2022; Sebaratnam et al., 2023).

Growing evidence shows a bidirectional relationship between gastrointestinal symptoms and psychological factors. Psychological comorbidities are common in patients with chronic gastroduodenal symptoms, and stress, anxiety, and depression have been found to trigger and exacerbate gastrointestinal symptoms (Palsson and Whitehead, 2013; Van Oudenhove et al., 2016; Wouters and Boeckxstaens, 2016; Woodhouse et al., 2017; Luo and Keefer, 2021; Raubinger et al., 2022; Knowles et al., 2023). The gut-brain axis, a complex neurohormonal pathway that facilitates communication between the brain and the gastrointestinal tract, plays a crucial role in this association (Carabotti et al., 2015; Keightley et al., 2015; Van Oudenhove et al., 2016; Weltens et al., 2018). Psychosocial factors are also significant determinants of treatment adherence, efficacy, healthcare utilization, and costs (Levy et al., 2006; Luo and Keefer, 2021). Psychological interventions have the potential to improve mental wellbeing and gastrointestinal symptoms in these patients (Palsson and Whitehead, 2013; Shoji et al., 2018; Rodrigues et al., 2021; Keefer et al., 2022; Singh et al., 2022). Therefore, early identification and management of psychological comorbidities are crucial for significant improvements in gastrointestinal symptoms and quality of life.

The growing recognition of the gut-brain axis and the adoption of a biopsychosocial framework have led to the recommendation of psychological assessments as a critical part of the standard care for managing chronic gastroduodenal symptoms (Van Oudenhove et al., 2016; Schmulson and Drossman, 2017; Luo and Keefer, 2021; van Tilburg et al., 2021). Clinicians routinely ask mental wellbeing questions when evaluating gastrointestinal patients; however, these questions are often asked informally without standardized psychometrics (Wu, 2012; Drossman et al., 2021; Law et al., 2023). Although validated and widely used mental health questionnaires exist, these have limitations when used within this patient population. For example, many questionnaires assessing depression and anxiety include questions on physical symptomatology, such as reduced appetite and disrupted sleep. However, in patients with chronic gastroduodenal symptoms, these physical manifestations may be primarily related to their gastrointestinal disorder. Therefore, these questions may not be a valid reflection of their mental health, potentially resulting in an overestimation of psychological concerns among these patients and inaccurate formulations of their symptomatology, which may lead to ineffective management (Luo and Keefer, 2021).

To date, no general mental health scale has been developed and validated among patients with chronic gastroduodenal symptoms. The ongoing improvement and validation of psychometrics within novel contexts and samples is recommended to increase the dependability and validity of the results while reducing patient burden and costs (Dima, 2018). Therefore, there is a need to develop a brief self-report scale specifically designed for use in patients with chronic gastroduodenal symptoms to ensure valid and reliable assessments of mental health. Here we describe the steps used to develop and validate a novel mental health scale, the Alimetry® Gut-Brain Wellbeing (AGBW) Survey for patients with chronic gastroduodenal symptoms.

## Methods

**Figure 1:**
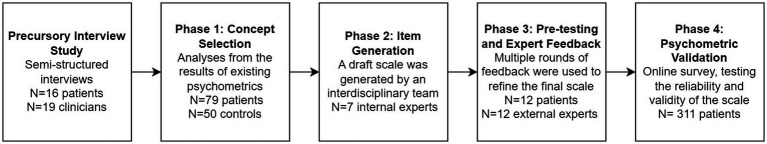
Flowchart of the phases used to develop the Alimetry Gut-Brain Wellbeing Survey.

As shown in Figure 1, the AGBW Survey was developed and validated in four mixed-methods phases, with guidance from the results from a precursory user needs interview study with patients with gastroduodenal DGBIs and gastroenterology clinicians (Law et al., 2023). The precursory interview study was conducted prior to the scale development phases, with results reported elsewhere (Law et al., 2023). Each phase involved co-design with gastroenterologists, psychogastroenterologists, and patients with chronic gastroduodenal symptoms to ensure face and content validity, comprehensibility, and acceptability.

### Phase 1: concept selection

The first phase aimed to identify the most important mental health concepts to include in the new scale, contextualized specifically for patients with chronic gastroduodenal symptoms. Valid general mental health constructs have already been conceptualized within psychometric development for general populations. Therefore, valid concepts were selected from existing mental health scales, using expert feedback and the analysis of psychometric data from a sample of patients with chronic gastroduodenal symptoms, to reduce the number of constructs to those most relevant and contextualized to the target patient population. The full methods for concept selection are provided in the Supplementary methods.

### Phase 2: item generation

An interdisciplinary panel of experts (including two health psychology researchers specializing in psychogastroenterology, a gastroenterologist, a gastrointestinal surgeon, a digital health translational researcher, and two bioengineers specializing in gastric electrophysiology) generated novel draft items based on the concepts identified from Phase 1.

### Phase 3: pre-testing and expert feedback

The draft scale was then pre-tested with a sample of patients with chronic gastroduodenal symptoms and reviewed by independent external experts to ensure the acceptability, clarity, comprehensibility, and content and face validity of the scale.

#### Sample

The pretesting sample consisted of 12 patients with chronic gastroduodenal symptoms (11 females; mean age = 33.3 years, age range = 20–55 years). All patients met the Rome IV criteria (The Rome Foundation, 2016) for functional dyspepsia, with 10 also having a coexisting diagnosis of chronic nausea and vomiting syndrome and nine also having a diagnosis of gastroparesis. The external experts comprised key opinion leaders in their respective areas, including nine gastroenterologists, one health psychologist specializing in psychogastroenterology, and two health psychology researchers. Patients and experts were recruited until data saturation.

#### Procedure

Experts and patients viewed the draft scale alongside images that showed potential implementation on a tablet interface. They answered a series of open-ended questions, including about the acceptability of the scale items, the utility of the scale in clinical practice, the ease of understanding and comprehension of the question wording, and whether the scale could be improved. This feedback was incorporated into the scale and sent back to respondents for further feedback. This process occurred until all reviewers were satisfied with the scale wording.

### Phase 4: psychometric validation

Psychometric validation of the scale was conducted using an anonymous, cross-sectional survey of patients with chronic gastroduodenal symptoms. All patients provided informed consent. Ethical approval was granted by the Auckland Health Research Ethics Committee Application (AH25798), and the trial was pre-registered at ANZCTR.org.au (ACTRN12623000385640).

#### Sample

Patients were recruited via convenience sampling through social media, clinic flyers, and clinic lists. Patients were included if they were over 18 years old and able to speak, read, and write fluently in English. Patients also had to meet the Rome IV criteria (The Rome Foundation, 2016) and/or have a self-reported clinical diagnosis for at least one of the following conditions: gastroparesis, functional dyspepsia, chronic nausea and vomiting syndrome, cyclic vomiting syndrome, rumination syndrome, cannabinoid hyperemesis syndrome, or a belching disorder. Clinician confirmation of diagnosis was not collected. Vulnerable participants and patients with self-induced vomiting or an eating disorder were excluded. Patients were recruited globally and efforts were made to ensure adequate recruitment across geographic regions, conditions, and genders.

#### Procedure

The anonymous survey was completed online via Qualtrics (Qualtrics, Provo, UT) and took approximately 15 min. Patients were presented with a demographics questionnaire and a battery of psychological questionnaires, including the AGBW Survey, presented in a randomized order. The survey ended with an optional feedback form about the scale and participants were provided with the option to enter a prize draw. Responses were collected between April 2023 and August 2023.

#### Measures

##### Demographics

Participants provided basic demographics, including age, gender, ethnicity, and country of residence. They also self-reported whether they had ever been diagnosed with a mental health issue.

##### Psychometrics

The following psychometrics were measured to assess convergent validity: the Patient Health Questionnaire 9 (PHQ-9) (Kroenke et al., 2001) to measure depression, the Generalized Anxiety Disorder 7 (GAD-7) (Spitzer et al., 2006) to measure anxiety, the Perceived Stress Scale 4 (PSS-4) (Cohen et al., 1983) to measure chronic stress, the Depression Anxiety and Stress Scale 21 (DASS-21) (Lovibond and Lovibond, 1995) to measure anxiety, depression, and stress in an integrated scale, and the Kessler Psychological Distress Scale (K-10) (Kessler et al., 2003) to measure total levels of distress.

The Big Five Inventory (BFI) extraversion subscale (John and Srivastava, 1999) and the Emotion Regulation Questionnaire (ERQ) (Gross and John, 2003) were measured to assess divergent validity. Lastly, concurrent validity was assessed using the Patient Assessment of Upper Gastrointestinal Disorders-Quality of Life (PAGI-QOL) (de la Loge et al., 2005), which measures the quality of life in patients with upper gastrointestinal disorders. These scales were chosen as they are some of the most commonly used questionnaires to assess depression, anxiety, stress, personality, and quality of life in healthcare settings.

##### AGBW survey feedback form

The optional feedback form asked participants to rate the AGBW Survey on a visual analogue scale for the following attributes; (1) how easy the questionnaire is to complete on a scale of 0 (very hard) to 100 (very easy), (2) how easy the questions are to understand on a scale of 0 (very hard) to 100 (very easy), and (3) how helpful they thought the scale would be for their gastric clinician to understand their mental wellbeing and provide them with more holistic care on a scale of 0 (very unhelpful) to 100 (very helpful). Participants were also asked to answer yes, no, or unsure to the question, “Would you like to see this questionnaire incorporated as part of routine assessment for your stomach symptoms, alongside medical testing?”

#### Statistical analysis

Data were analyzed using IBM SPSS Statistics v29. A *p*-value of 0.05 was considered statistically significant. Partial responses were included in the study as long as the patient had completed at least the demographics questions and the first four questions (the depression subscale) of the AGBW Survey. As a result, the validity and reliability calculations vary in terms of the number of respondents included, with a minimum of *N* = 295 within each calculation.

##### Confirmatory factor analysis

Confirmatory factor analysis with the maximum likelihood estimation method was conducted using IBM SPSS AMOS v26 using a three-factor model, splitting the depression, stress, and anxiety questions into separate factors. The model’s goodness of fit was evaluated using multiple indices: chi-square/degree of freedom (*χ^2^/df*), the normed fit index (NFI), the comparative fit index (CFI), the Tucker Lewis Index (TLI), the standardized root mean square residual (SRMR), and the root mean square error of approximation (RMSEA). An acceptable model of fit was predefined as a *χ2/df* < 5, an NFI > 0.95, a CFI > 0.90, and TLI > 0.95, an SRMR < 0.08, and an RMSEA < 0.08 (Hu and Bentler, 1999; Yusoff et al., 2021; Kline, 2023).

##### Reliability

Cronbach’s alpha coefficients (α) were calculated to examine the internal consistency reliability of the subscale and total scores (DeVon et al., 2007). A value of *α* > 0.70 indicates acceptable reliability, *α* > 0.80 ideal reliability, and *α* > 0.90 excellent reliability (Nunnally and Bernstein, 1994; DeVon et al., 2007; Terwee et al., 2007; Boateng et al., 2018). Inter-item correlations and corrected item-total correlations were calculated to assess the correlations between the scale items and the correlations between each item and the subscale scores/total score without that item, respectively. A value of *r* > 0.30 indicates good consistency between the scale items (for inter-item correlations) and the subscale/total scores (for item-total correlations) (DeVon et al., 2007; Boateng et al., 2018).

##### Validity

To demonstrate good construct and criterion validity for the subscale and total scores, at least or equal to 75% of the hypotheses below for convergent, divergent, concurrent, and known-groups validity were required to be met for each subscale/total score (Terwee et al., 2007; Abma et al., 2016).

The convergent validity of the AGBW depression subscale was assessed using Pearson’s correlation coefficients with the PHQ-9 total score and the depression subscale score of the DASS-21; the AGBW stress subscale was compared with the PSS-4 total score and the DASS-21 stress subscale score; the AGBW anxiety subscale was compared against the GAD-7 total score and the DASS-21 anxiety subscale score; and lastly, the AGBW total score was compared against the K-10 total score. A value of *r* > 0.50 indicates good convergent validity and *r* > 0.30 acceptable convergent validity (DeVon et al., 2007; Carlson and Herdman, 2012; Abma et al., 2016).

Divergent validity was analyzed using Pearson’s correlation coefficients between the AGBW subscale and total scores, and the BFI extraversion subscale score, the ERQ cognitive reappraisal subscale score, and the ERQ expressive suppression subscale score. Divergent validity was achieved if these correlation coefficients were weaker than the correlations with the scales used for convergent validity (DeVon et al., 2007; Rönkkö and Cho, 2022).

Concurrent validity was analyzed using Pearson’s correlation coefficients between the AGBW subscale and total scores and the PAGI-QoL total score and psychological wellbeing and distress subscale score. Concurrent validity was achieved if these correlation coefficients were positive and statistically significant; with *r* > 0.50 indicating strong, and *r* = 0.30–0.50 moderate, and *r* < 0.30 weak evidence.

Known groups validity was analyzed using one-tailed independent samples *t*-tests between groups who theoretically should have different scores on the scale (Boateng et al., 2018). Patients with a previous mental health diagnosis were expected to have significantly higher average scores on the AGBW subscale and total scores than those without a previous mental health diagnosis. Females were also expected to have significantly higher average subscale and total scores than males (Parker and Brotchie, 2010; Bouchoucha et al., 2013; Altemus et al., 2014; Jalnapurkar et al., 2018).

## Results

### Phase 1: concept selection

**Figure 2:**
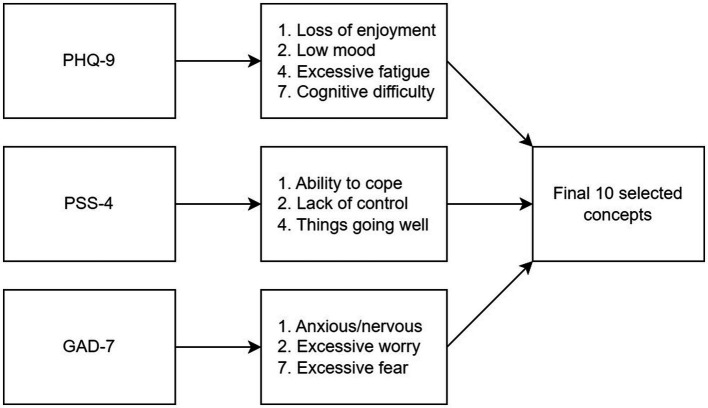
Flowchart showing the concepts selected from the original psychometrics.

Ten key concepts were identified as the most important indicators of mental health in this sample of patients with chronic gastroduodenal symptoms. These concepts were derived from specific items across three scales: items 1, 2, 4, and 7 of the PHQ-9; items 1, 2, and 3 of the PSS-4; and items 1, 2, and 7 of the GAD-7 (Figure 2). Further detail about how these 10 concepts were selected is provided in the Supplementary results.

### Phase 2: item generation

The expert panel drafted 10 novel closed-ended questions to cover the 10 concepts identified in Phase 1. A 5-point response format was chosen based on recommendations that reliability increases with more scale points for unipolar items, with diminishing returns after 5 points (Fabrigar and Ebel-Lam, 2007; Boateng et al., 2018). Similar to the PHQ-9 and GAD-7, a recall period of 2 weeks was chosen as this matches the criteria in the Diagnostic and Statistical Manual of Mental Health Disorders (5th Ed., DSM-5) (American Psychiatric Association, 2013) for depression and anxiety and is recommended by the FDA due to increased recall bias beyond 2 weeks (U.S. Department of Health and Human Services Food and Drug Administration, 2009).

Both patients and clinicians in the precursory interview study (Law et al., 2023) iterated the importance of prefacing patients before mental health assessments to explain why this information is being gathered and to reduce stigma and reassure them that this data will not be used to dismiss medical care. Based on these recommendations, the expert panel drafted a short preface explaining how this scale can help clinicians develop a more holistic understanding of the patient’s condition, enabling them to deliver a more tailored management plan. The preface also communicates how the scale is not diagnostic and cannot be used to attribute their gastrointestinal symptoms to their mental health.

Lastly, an opt-out option was included at the end of the preface to allow patients to decline the survey. The responses from the precursory interviews (Law et al., 2023) determined this to be an essential addition for patients concerned about how their clinician might misuse or misinterpret their responses. A follow-up optional, open-ended question is provided for patients to leave a comment about why they have chosen not to answer this survey. This question can help clinicians understand why the patient declined to answer, which may be relevant to their future care.

### Phase 3: pre-testing and expert feedback

Two rounds of feedback were gathered and used to refine the final scale. Overall, the scale had high content and face validity and was seen as acceptable, easy to understand and complete, and useful to help clinicians further understand a patient’s condition and aid in developing tailored management. Both patients and clinicians were supportive of the use of the scale within clinical care for patients with chronic gastroduodenal symptoms.

Based on the feedback, the wording of the preface and some of the scale items were edited to ensure clarity and ease of answering. The preface was expanded to include more information about the gut-brain axis to help patients further understand why they are being asked about their mental health. Lastly, an optional open-ended question was added to the end of the scale to allow patients to include additional comments regarding their mental wellbeing, enabling their clinician to develop a more comprehensive understanding of the survey results.

### The final scale: the Alimetry Gut-Brain Wellbeing Survey

The final scale (see Supplementary file) consists of a patient preface, 10 closed-ended questions, and an optional 11th open-ended question. The patient preface explains to patients why these questions are being asked and how the data is being used. The 10 closed questions ask patients to rate how often they have felt or behaved in a certain way over the last 2 weeks on a scale of 0 (none of the time) to 4 (all of the time). Items 5 and 7 are written in a positive frame and must be reverse-coded. The scores from each question can be totaled to create a total gut-brain wellbeing score (out of 40). Three subscales can also be calculated; a depression subscale score made up of the total of questions 1–4 (out of 16), a stress subscale score made up of the total of questions 5–7, after reverse coding questions 5 and 7 (out of 12), and an anxiety subscale score made up of the total of questions 8–10 (out of 12). Higher scores indicate worse mental health. The scale concludes with an optional open-ended question asking patients to add any further comments about their mental wellbeing.

### Phase 4: psychometric validation

#### Sample characteristics

A total of 311 participants completed the validation survey (mean age = 38.40 years, *SD* = 14.21, range = 18–76 years). Most respondents were female, white, and from the USA or British Commonwealth (Table 1). The Other countries included France (*n* = 1), Chile (*n* = 1), the Netherlands (*n* = 1), Ireland (*n* = 1), and Puerto Rico (*n* = 1). There was large overlap between the Rome diagnoses met by patients, with most patients meeting the Rome IV criteria for functional dyspepsia and chronic nausea and vomiting syndrome. Only 14% of respondents met the Rome IV criteria for only one gastroduodenal DGBI, with the majority (*n* = 186, 60%) meeting the criteria for three or more. Most patients had a self-reported previous diagnosis of anxiety or depression, with 74% self-reporting a previous diagnosis of any mental health disorder.

**Table 1:** Demographic characteristics of the survey respondents (*N* = 311).

	*n* (%)
**Gender**	
Female	262 (84%)
Male	38 (12%)
Gender diverse	11 (4%)
**Country**	
United States	120 (39%)
New Zealand	89 (29%)
Australia	43 (14%)
Canada	29 (9%)
United Kingdom	25 (8%)
Other	5 (2%)
**Ethnicity**	
White	259 (83%)
Black	5 (2%)
Asian	13 (4%)
Hispanic	14 (5%)
Māori/Pasifika	12 (4%)
Other	8 (3%)
**Rome IV Criteria** ^a^	
Functional dyspepsia	260 (84%)
Chronic nausea and vomiting syndrome	246 (79%)
Cyclic vomiting syndrome	119 (38%)
Cannabinoid hyperemesis syndrome	7 (2%)
Rumination syndrome	115 (37%)
Belching disorder	126 (41%)
Diagnosis of gastroparesis	168 (54%)
**Previous mental health diagnoses**	
Depression	182 (59%)
Anxiety	192 (62%)
Other	62 (20%)
None	82 (26%)
Prefer not to say	4 (1%)

n, number of respondents in that category; %, percentage of respondents in that category; ^a^Rome IV criteria diagnosis is based on meeting the symptom criteria only; respondents can meet more than one Rome IV diagnosis.

#### Descriptive statistics

**Figure 3:**
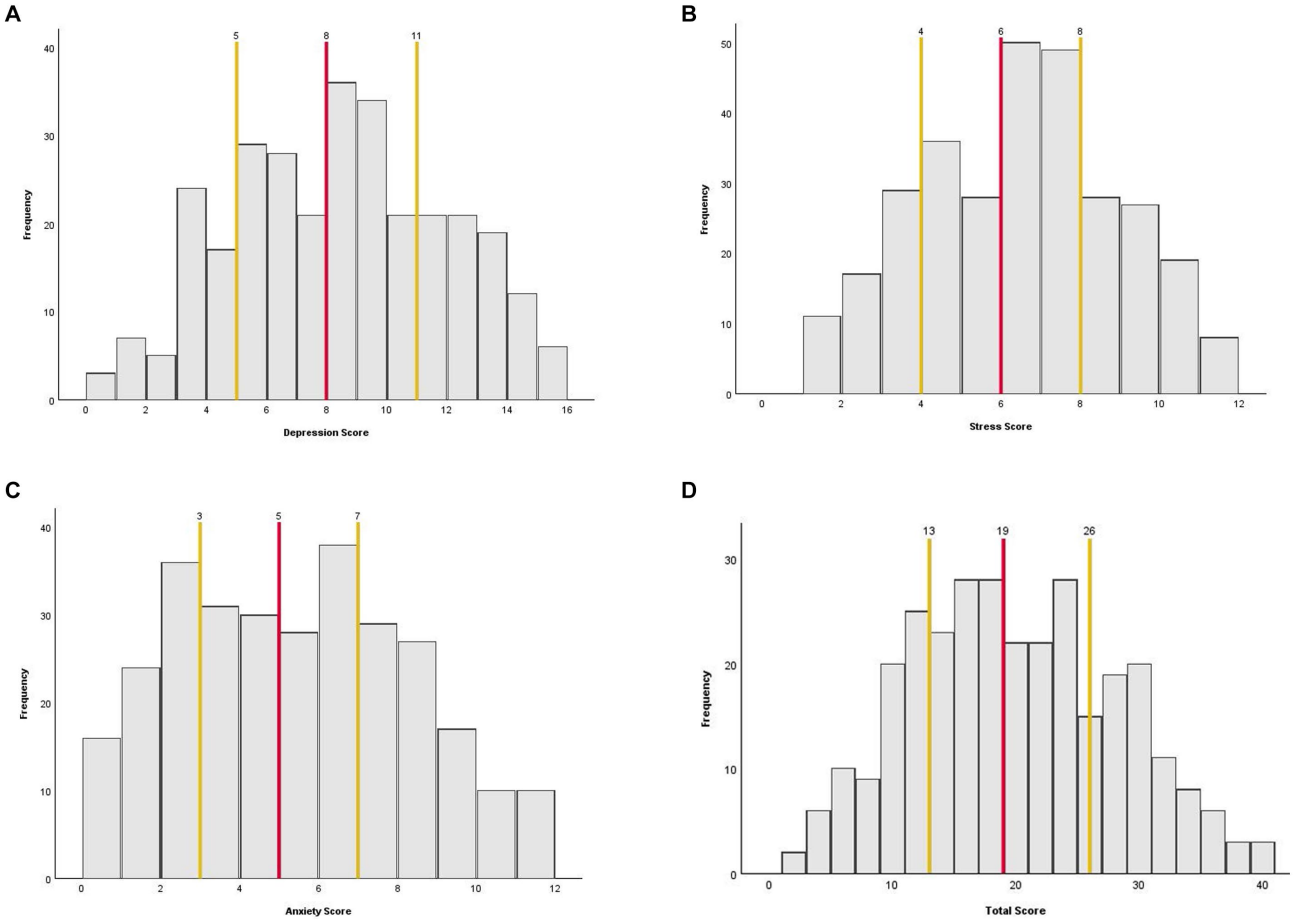
Histograms showing the distribution of the AGBW Survey subscale and total scores across the sample. **(A)** Depression subscale, **(B)** stress subscale, **(C)** anxiety subscale, and **(D)** total score. The red line indicates the median score and the yellow lines indicate the interquartile ranges.

The 10 questions took participants an average of 87 s to complete (*IQR* = 58–107 s). As shown in Table 2, the full range of subscale scores available were used, except for the stress subscale, where no patient scored a 0/12. However, all individual questions used the whole range of answers (0–4). All three subscale scores and the total score were normally distributed, as shown by the lack of skew and kurtosis in Table 2 and the scale distributions in Figure 3. The mean and median scores of each subscale and the total score were in the middle of the scale range, further demonstrating normality.

**Table 2:** Descriptive statistics of the AGBW Survey’s subscales and total scores.

Scale	*N*	*M*	*SD*	Range	Skewness	Kurtosis	*α*
Depression subscale	311	8.11	3.71	0–16	0.08	−0.69	0.81
Stress subscale	308	6.09	2.62	1–12	0.07	−0.60	0.72
Anxiety subscale	308	5.20	3.19	0–12	0.30	−0.72	0.86
Total score	308	19.40	8.37	2–40	0.16	−0.66	0.90

N, number of respondents; M, mean; SD, standard deviation; α, Cronbach’s alpha coefficient.

#### Confirmatory factor analysis

The confirmatory factor analysis revealed that the three-factor model, splitting the depression, stress, and anxiety questions into separate factors, had a good fit, meeting all predefined acceptability criteria (*χ^2^*_(32)_ = 76.00, *p* < 0.001, *χ^2^/df* = 2.37, NFI = 0.95, CFI = 0.97, TLI = 0.96, SRMR = 0.04, RMSEA = 0.07 [0.05–0.09]).

#### Reliability

As shown in Table 2, the Cronbach’s *α* coefficients demonstrated excellent internal consistency reliability for the AGBW Survey total score, good internal consistency reliability for the anxiety and depression subscale scores, and acceptable internal consistency reliability for the stress subscale score. In the total score and all subscales, eliminating additional items resulted in no substantial increases in reliability. The inter-item correlations ranged from *r* = 0.31–0.75, indicating good consistency between the individual items. The corrected item-total correlations ranged from *r* = 0.53–0.78, indicating good consistency between the individual items and the subscale and total scores.

#### Validity

##### Convergent validity

Most of the correlations between the AGBW Survey’s subscale scores and total scores and the scales used to assess convergent validity showed significant correlations with large effect sizes over *r* = 0.50 (Table 3), indicating good convergent validity. However, the correlation between the AGBW stress subscale and the DASS-21 stress subscale was just under *r* = 0.50, indicating acceptable convergent validity despite the high correlation with the PSS-4.

**Table 3:** Pearson correlation coefficients between the AGBW Survey scores and comparative questionnaires used for validity testing.

		Depression subscale	Stress subscale	Anxiety subscale	Total score
Convergent validity	PHQ-9 total	0.77*			
	PSS-4 total		0.80*		
	GAD-7 total			0.78*	
	DASS-21 depression subscale	0.70*			
	DASS-21 stress subscale		0.46*		
	DASS-21 anxiety subscale			0.57*	
	K-10 total				0.87*
Divergent validity	BFI extraversion subscale	−0.26*	−0.27*	−0.20*	−0.28*
	ERQ cognitive reappraisal subscale	−0.32*	−0.32*	−0.24*	−0.34*
	ERQ expressive suppression subscale	0.22*	0.19*	0.20*	0.23*
Concurrent validity	PAGI-QoL total	0.64*	0.52*	0.55*	0.66*
	PAGI-QoL psychological wellbeing and distress subscale	0.74*	0.64*	0.68*	0.79*

PHQ-9, Patient Health Questionnaire 9; PSS-4, Perceived Stress Scale 4; GAD-7, Generalized Anxiety Disorder 7; DASS-21, Depression Anxiety and Stress Scale 21; K-10, Kessler Psychological Distress Scale; BFI, Big Five Inventory; ERQ, Emotion Regulation Questionnaire; PAGI-QoL, Patient Assessment of Upper Gastrointestinal Disorders-Quality of Life; * denotes significance at *p* < 0.05.

##### Divergent validity

The correlations between the AGBW subscale and total scores and the BFI and ERQ scores all demonstrated small effect sizes, which were considerably lower than the convergent validity correlations (Table 3), demonstrating successful divergent validity.

##### Concurrent validity

All correlations between the AGBW subscale and total scores and the PAGI-QoL total scores and psychological wellbeing and distress subscale scores were statistically significant and over *r* = 0.50, demonstrating large effect sizes and therefore providing strong evidence of concurrent validity (Table 3).

##### Known groups validity

As shown in Table 4, there was a significant difference between those with and without any previous mental health diagnosis, with very large effect sizes. Those with a previous mental health diagnosis scored on average higher on all three subscale scores and the AGBW Survey total score than those who did not have a previous mental health diagnosis, indicating good known groups validity.

**Table 4:** Results from the independent samples *t*-tests used for known groups validity of the AGBW Survey.

		Depression subscale	Stress subscale	Anxiety subscale	Total score
Previous mental health diagnosis	Yes, *M* (SD)	9.18 (3.39)	6.74 (2.40)	6.07 (3.03)	22.00 (7.50)
	No, *M* (SD)	5.38 (2.97)	4.44 (2.39)	2.99 (2.37)	12.71 (6.40)
	*p*-value	<0.001*	<0.001*	<0.001*	<0.001*
	Cohen’s *d*	1.16	0.96	1.07	1.28
Gender	Female, *M* (SD)	7.96 (3.65)	6.06 (2.61)	5.16 (3.26)	19.18 (8.36)
	Male, *M* (SD)	8.42 (3.96)	6.14 (2.81)	5.03 (2.69)	19.54 (8.58)
	*p*-value	0.237	0.437	0.405	0.405
	Cohen’s *d*	0.12	0.03	0.04	0.04

M, mean; SD, standard deviation; *p*-values were calculated using independent samples *t*-tests; * denotes significance at *p* < 0.05.

Additionally, independent samples t-tests showed that those patients who had a previous depression diagnosis had significantly higher scores on the AGBW depression subscale (*M* = 9.44, *SD* = 3.46), than those who did not have a previous depression diagnosis (*M* = 6.31, *SD* = 3.19, *p* < 0.001, *d* = 0.93), with a large effect size. Similarly, those who had a previous diagnosis of an anxiety disorder had higher scores on the AGBW anxiety subscale (*M* = 6.32, *SD* = 3.03) than those who did not have a previous anxiety diagnosis (*M* = 3.45, *SD* = 2.52, *p* < 0.001, *d* = 1.01), with a large effect size.

In contrast, there were no significant differences between males and females for any of the subscale or total scores (Table 4), as was hypothesized in the predefined acceptability criteria (Parker and Brotchie, 2010; Bouchoucha et al., 2013; Altemus et al., 2014; Jalnapurkar et al., 2018).

#### Feedback form

Two hundred and forty patients completed the optional feedback form. The scale questions were rated as easy to complete and easy to understand, with an average score of 85.13 (*SD* = 18.48) and 85.77 (*SD* = 17.51) out of 100, respectively. The patients also rated the questions as helpful for their gastric clinician to understand their mental wellbeing and provide them with more holistic care, with an average score of 70.24 (*SD* = 26.36) out of 100. Lastly, although 33% were unsure if they would like to see these questions incorporated as part of the routine assessment for their stomach symptoms, only 10% answered no, and 57% indicated yes, suggesting the overall acceptability of these questions as part of routine testing.

## Discussion

This research used a multi-phase, mixed-methods process, incorporating co-design with patients and clinicians to develop and validate a brief, novel mental health scale for patients with chronic gastroduodenal symptoms.

The AGBW Survey was developed to be a novel addition to the existing pool of psychometrics for patients with chronic gastroduodenal symptoms, complementing other mental wellbeing tools that measure gastrointestinal symptom-specific anxiety (Labus et al., 2004) and quality of life (de la Loge et al., 2005). Due to the high presence of psychological comorbidities (Shoji et al., 2018; Luo and Keefer, 2021) and clinical recommendations for routine psychological assessments in these patients (Van Oudenhove et al., 2016; Keefer et al., 2018; Luo and Keefer, 2021), clinicians have typically relied on general mental health scales. However, these assessments are not contextualized for use within patients with chronic gastroduodenal symptoms and may potentially exaggerate the reporting of mental health issues due to the wording of items that emphasize physical symptomatology (Luo and Keefer, 2021). In contrast, the AGBW Survey is designed to focus more specifically on mental health concepts relevant to patients with chronic gastroduodenal symptoms.

Furthermore, the AGBW Survey is multidimensional and combines assessments of depression, anxiety, and stress into a single, brief scale, with confirmatory factor analysis supporting the presence of this three-factor model. This scale allows a quick assessment of a patient’s general wellbeing as well as more specific dimensions of common mental health issues. This scale’s brief nature reduces the clinician and patient burden associated with psychological assessment batteries, which is particularly important as gastroenterology clinicians often report time constraints as the key reason for not regularly assessing a patient’s mental wellbeing, despite being aware of its importance (Law et al., 2023). The AGBW Survey is intended to complement routine medical tests, such as body surface gastric mapping (Gharibans et al., 2022; O’Grady et al., 2023), to provide more integrated evaluations and management plans for patients with chronic gastroduodenal symptoms. Research has demonstrated that incorporating psychological support for patients with chronic gastroduodenal symptoms as part of multidisciplinary care leads to better care and more effective symptom management (Kruimel et al., 2015; Riehl et al., 2019; Basnayake et al., 2020; Bray et al., 2022). Therefore, the results from the AGBW Survey could prompt clinicians to consider incorporating psychological referrals or interventions into the patient’s care plan alongside traditional medical care.

The AGBW Survey demonstrated excellent reliability and good validity, including strong correlations with existing mental health measures, indicating its suitability for clinical use. The scale also successfully discriminated between patients with and without a self-reported mental health diagnosis. Similar results were also seen for the three individual subscale scores. However, the stress subscale did show lower, but still acceptable, internal consistency reliability and convergent validity. This finding is likely related to the fact that the stress subscale included two reverse-coded items, which, by nature, can lead to lower reliability (Carlson et al., 2011; Weijters et al., 2013). Additionally, the DASS-21 stress subscale was only moderately correlated with both the AGBW stress subscale and the PSS-4, suggesting that the lower convergent validity may be attributable to the DASS-21, rather than the AGBW subscale.

The scale’s psychometric properties demonstrated a normal distribution with little to no skew. Generally, psychometrics are positively skewed, with most respondents scoring on the lower end (Blanca et al., 2013; Bono et al., 2017). However, patients with chronic gastroduodenal symptoms often experience higher psychological comorbidities than the general population (Tse et al., 2010; Wu, 2011; Wouters and Boeckxstaens, 2016; Woodhouse et al., 2017), which may account for the high scores and lack of skew. This finding was further emphasized by the high number of participants with a previous mental health diagnosis, which further highlights the importance of routinely assessing mental wellbeing in this patient population.

Contrary to our hypothesis, the total and subscale scores were unable to discriminate between males and females, which could be attributed to the low number of males recruited. Males are less likely than females to have a functional gastrointestinal disorder (Sperber et al., 2021) and participate in online survey-based research (Becker, 2022; Wu et al., 2022), so the limited male representation is unsurprising, even despite researcher efforts to boost male participation. Evidence suggests that males with functional gastrointestinal disorders are less depressed, anxious, and stressed than females, regardless of their symptom severity (Addolorato et al., 2008; Bouchoucha et al., 2013). However, in the current study, males had relatively high scores on the scale, potentially due to self-selection bias. Despite this, the pre-defined acceptability criteria, which were based on existing guidelines for questionnaire validation (Terwee et al., 2007; Abma et al., 2016), detailed that at least or equal to 75% of the validity hypotheses must be met for each subscale and the total score to demonstrate good construct and criterion validity. Even with the lack of successful gender discrimination, 90% of the validity hypotheses were met, indicating successful validation of the AGBW Survey.

A novel aspect of this scale is the addition of the patient preface, opt-out option, and final open-ended question. These were important additions suggested by clinicians and patients during the precursory interviews (Law et al., 2023) and feedback phase to increase the scale’s acceptability and usability, and decrease stigma, which was noted as a potential concern. In particular, the open-ended question allows patients to provide valuable context to their survey responses, providing clinicians with important additional information that can be used for targeted clinical management plans and reducing the chance of the patient’s symptoms being dismissed as exclusively psychological.

Strengths of this scale development process include the co-development with patients, clinicians, and interdisciplinary experts throughout every phase, which ensured the development of an acceptable, understandable, useful, and clinically relevant scale. Furthermore, due to the international approach, the samples included patients from diverse countries and health contexts. However, most patients were from Western countries and self-identified as ethnically white, which may restrict the generalizability of these results to other cultural contexts. Previous research has shown the invariance of existing mental health scales across ethnicities, such as the PHQ-9 (Galenkamp et al., 2017; Patel et al., 2019) and GAD-7 (Sriken et al., 2022). Therefore, it is expected the AGBW Survey should have a similar ethnic invariance. With the inclusion of 7% US ethnic minorities, we expect the results to be generalizable in the US, although additional ethnicity contexts were not addressed. Furthermore, the scale is currently only available in English and still needs to be translated and validated in other languages.

Additionally, as the validation survey was cross-sectional, we could not assess the scale’s predictive validity. Research is currently underway to evaluate whether the combination of the results from the AGBW Survey and physiological tests, such as body surface gastric mapping (Gharibans et al., 2022; O’Grady et al., 2023), can help predict which patients benefit from integrated care. This research will further inform the scale’s clinical utility as an aid to guide case formulation and clinical management decisions. Furthermore, future research should work to establish population norms and develop scoring systems to aid in interpreting individual scores.

### Summary

The AGBW Survey was developed using a multi-phase, co-design process that included precursory interviews, data from existing psychometrics, consensus from an interdisciplinary panel of experts, and feedback from clinicians, patients, and key opinion leaders in gastroenterology. This research demonstrates that the AGBW Survey is a well-accepted, valid, and reliable scale for assessing mental health in patients with chronic gastroduodenal symptoms. This scale combines assessments of depression, stress, and anxiety, using items contextualized for patients with chronic gastroduodenal symptoms, allowing a brief assessment of a patient’s mental health. While not designed for diagnostic purposes, the AGBW Survey can be incorporated into routine clinical testing to complement existing physiological tests to provide more integrated evaluations and management plans. Moreover, its utility extends to research contexts, where it can be used to assess a patient’s mental health at baseline or evaluate changes over time in relation to symptoms, disease progression, or interventions.

## Data availability statement

The raw data supporting the conclusions of this article will be made available by the authors, without undue reservation.

## Ethics statement

The studies involving humans were approved by Auckland Health Research Ethics Committee Application (AH25798). The studies were conducted in accordance with the local legislation and institutional requirements. The participants provided their written informed consent to participate in this study.

## Author contributions

ML: Conceptualization, Data curation, Formal analysis, Investigation, Methodology, Project administration, Visualization, Writing – original draft, Writing – review & editing. IP: Conceptualization, Investigation, Methodology, Writing – review & editing. GH: Conceptualization, Investigation, Validation, Writing – review & editing. GSe: Conceptualization, Investigation, Writing – review & editing. GSc: Writing – review & editing. KS: Writing – review & editing. CV: Writing – review & editing. PD: Writing – review & editing. CD: Investigation, Writing – review & editing. I-HH: Writing – review & editing. SK: Writing – review & editing. AG: Supervision, Writing – review & editing. GO'G: Conceptualization, Funding acquisition, Resources, Supervision, Writing – review & editing. CA: Conceptualization, Investigation, Methodology, Supervision, Writing – review & editing. SC: Conceptualization, Formal analysis, Investigation, Methodology, Resources, Supervision, Writing – review & editing.

## References

[ref1] Abma I. L. Rovers M. van der Wees P. J. (2016). Appraising convergent validity of patient-reported outcome measures in systematic reviews: constructing hypotheses and interpreting outcomes. BMC. Res. Notes 9:226. doi: 10.1186/s13104-016-2034-2 27094345 PMC4837507

[ref2] Addolorato G. Mirijello A. D’Angelo C. Leggio L. Ferrulli A. Abenavoli L.. (2008). State and trait anxiety and depression in patients affected by gastrointestinal diseases: psychometric evaluation of 1641 patients referred to an internal medicine outpatient setting. Int. J. Clin. Pract. 62, 1063–1069. doi: 10.1111/j.1742-1241.2008.01763.x 18422970

[ref3] Altemus M. Sarvaiya N. Neill E. C. (2014). Sex differences in anxiety and depression clinical perspectives. Front. Neuroendocrinol. 35, 320–330. doi: 10.1016/j.yfrne.2014.05.004, PMID: 24887405 PMC4890708

[ref4] American Psychiatric Association (2013). Diagnostic and statistical manual of mental disorders, vol. 5.

[ref5] Basnayake C. Kamm M. A. Salzberg M. R. Wilson-O’Brien A. Stanley A. Thompson A. J. (2020). Delivery of care for functional gastrointestinal disorders: a systematic review. J. Gastroenterol. Hepatol. 35, 204–210. doi: 10.1111/jgh.14830 31411755

[ref6] Becker R. (2022). Gender and survey participation: an event history analysis of the gender effects of survey participation in a probability-based multi-wave panel study with a sequential mixed-model design. Methods Data Anal. 16:30. doi: 10.12758/mda.2021.08

[ref7] Blanca M. J. Arnau J. López-Montiel D. Bono R. Bendayan R. (2013). Skewness and kurtosis in real data samples. Methodology 9, 78–84. doi: 10.1027/1614-2241/a000057

[ref8] Boateng G. O. Neilands T. B. Frongillo E. A. Melgar-Quiñonez H. R. Young S. L. (2018). Best practices for developing and validating scales for health, social, and behavioral research: a primer. Front. Public Health 6:149. doi: 10.3389/fpubh.2018.00149, PMID: 29942800 PMC6004510

[ref9] Bono R. Blanca M. J. Arnau J. Gómez-Benito J. (2017). Non-normal distributions commonly used in health, education, and social sciences: a systematic review. Front. Psychol. 8:1602. doi: 10.3389/fpsyg.2017.01602, PMID: 28959227 PMC5603665

[ref10] Bouchoucha M. Hejnar M. Devroede G. Babba T. Bon C. Benamouzig R. (2013). Anxiety and depression as markers of multiplicity of sites of functional gastrointestinal disorders: a gender issue? Clin. Res. Hepatol. Gastroenterol. 37, 422–430. doi: 10.1016/j.clinre.2012.10.011 23270854

[ref11] Bray N. A. Koloski N. A. Jones M. P. Do A. Pang S. Coombes J. S.. (2022). Evaluation of a multidisciplinary integrated treatment approach versus standard model of care for functional gastrointestinal disorders (FGIDS): a matched cohort study. Dig. Dis. Sci. 67, 5593–5601. doi: 10.1007/s10620-022-07464-1, PMID: 35362835 PMC9652261

[ref12] Carabotti M. Scirocco A. Maselli M. A. Severi C. (2015). The gut-brain axis: interactions between enteric microbiota, central and enteric nervous systems. Ann. Gastroenterol. Hepatol. 28, 203–209. PMC4367209 25830558

[ref13] Carlson K. D. Herdman A. O. (2012). Understanding the impact of convergent validity on research results. Organ. Res. Methods 15, 17–32. doi: 10.1177/1094428110392383

[ref14] Carlson M. Wilcox R. Chou C.-P. Chang M. Yang F. Blanchard J.. (2011). Psychometric properties of reverse-scored items on the CES-D in a sample of ethnically diverse older adults. Psychol. Assess. 23, 558–562. doi: 10.1037/a0022484, PMID: 21319906 PMC3115428

[ref15] Cohen S. Kamarck T. Mermelstein R. (1983). A global measure of perceived stress. J. Health Soc. Behav. 24, 385–396. doi: 10.2307/2136404 6668417

[ref16] de la Loge C. Trudeau E. Marquis P. Kahrilas P. Stanghellini V. Talley N. J.. (2005). Cross-cultural development and validation of a patient self-administered questionnaire to assess quality of life in upper gastrointestinal disorders: the PAGI-QOL. Qual. Life Res. 13, 1751–1762. doi: 10.1007/s11136-004-8751-3, PMID: 15651545

[ref17] DeVon H. A. Block M. E. Moyle-Wright P. Ernst D. M. Hayden S. J. Lazzara D. J.. (2007). A psychometric toolbox for testing validity and reliability. J. Nurs. Scholarsh. 39, 155–164. doi: 10.1111/j.1547-5069.2007.00161.x 17535316

[ref18] Dima A. L. (2018). Scale validation in applied health research: tutorial for a 6-step R-based psychometrics protocol. Health Psychol. Behav. Med. 6, 136–161. doi: 10.1080/21642850.2018.1472602, PMID: 34040826 PMC8133536

[ref19] Drossman D. A. (2016). Functional gastrointestinal disorders: history, pathophysiology, clinical features and Rome IV. Gastroenterology 150, 1262–1279.e2. doi: 10.1053/j.gastro.2016.02.032, PMID: 27144617

[ref20] Drossman D. A. Chang L. Deutsch J. K. Ford A. C. Halpert A. Kroenke K.. (2021). A review of the evidence and recommendations on communication skills and the patient-provider relationship: a Rome foundation working team report. Gastroenterology 161, 1670–1688.e7. doi: 10.1053/j.gastro.2021.07.037, PMID: 34331912

[ref21] Fabrigar L. R. Ebel-Lam A. (2007). “Questionnaires” in Encyclopedia of measurement and statistics. ed. Salkind N. J., (SAGE Publications, Inc). 808–812.

[ref22] Galenkamp H. Stronks K. Snijder M. B. Derks E. M. (2017). Measurement invariance testing of the PHQ-9 in a multi-ethnic population in Europe: the HELIUS study. BMC Psychiatry 17:349. doi: 10.1186/s12888-017-1506-9, PMID: 29065874 PMC5655879

[ref23] Gharibans A. A. Calder S. Varghese C. Waite S. Schamberg G. Daker C.. (2022). Gastric dysfunction in patients with chronic nausea and vomiting syndromes defined by a noninvasive gastric mapping device. Sci. Transl. Med. 14:eabq3544. doi: 10.1126/scitranslmed.abq3544, PMID: 36130019 PMC10042458

[ref24] Gross J. J. John O. P. (2003). Individual differences in two emotion regulation processes: implications for affect, relationships, and well-being. J. Pers. Soc. Psychol. 85, 348–362. doi: 10.1037/0022-3514.85.2.348, PMID: 12916575

[ref25] Hu L. Bentler P. M. (1999). Cutoff criteria for fit indexes in covariance structure analysis: conventional criteria versus new alternatives. Struct. Equ. Modeling 6, 1–55. doi: 10.1080/10705519909540118

[ref26] Jalnapurkar I. Allen M. Pigott T. (2018). Sex differences in anxiety disorders: a review. J Psychiatry Depress Anxiety 4, 1–4. doi: 10.24966/PDA-0150/100012

[ref27] John O. P. Srivastava S. (1999). “The big five trait taxonomoy: history, measurement, and theoretical perspectives” in Handbook of personality: theory and research. eds. Pervin L. A. John O. P., vol. 2 (New York city: Guilford Press), 102–138.

[ref28] Keefer L. Ballou S. K. Drossman D. A. Ringstrom G. Elsenbruch S. Ljótsson B. (2022). A Rome working team report on brain-gut behavior therapies for disorders of gut-brain interaction. Gastroenterology 162, 300–315. doi: 10.1053/j.gastro.2021.09.015 34529986

[ref29] Keefer L. Palsson O. S. Pandolfino J. E. (2018). Best practice update: incorporating psychogastroenterology into management of digestive disorders. Gastroenterology 154, 1249–1257. doi: 10.1053/j.gastro.2018.01.045, PMID: 29410117

[ref30] Keightley P. C. Koloski N. A. Talley N. J. (2015). Pathways in gut-brain communication: evidence for distinct gut-to-brain and brain-to-gut syndromes. Aust. N. Z. J. Psychiatry 49, 207–214. doi: 10.1177/0004867415569801, PMID: 25710826

[ref31] Kessler R. C. Barker P. R. Colpe L. J. Epstein J. F. Gfroerer J. C. Hiripi E.. (2003). Screening for serious mental illness in the general population. Arch. Gen. Psychiatry 60, 184–189. doi: 10.1001/archpsyc.60.2.184 12578436

[ref32] Kline R. B. (2023). Principles and practice of structural equation modeling. New York city: Guilford Publications.

[ref33] Knowles S. R. Skvarc D. Ford A. C. Palsson O. S. Bangdiwala S. I. Sperber A. D.. (2023). Negative impact of disorders of gut-brain interaction on health-related quality of life: results from the Rome foundation global epidemiology survey. Gastroenterology 164, 655–68.e10. doi: 10.1053/j.gastro.2022.12.009, PMID: 36565940

[ref34] Kroenke K. Spitzer R. L. Williams J. B. (2001). The PHQ-9: validity of a brief depression severity measure. J. Gen. Intern. Med. 16, 606–613. doi: 10.1046/j.1525-1497.2001.016009606.x, PMID: 11556941 PMC1495268

[ref35] Kruimel J. Leue C. Winkens B. Marcus D. Schoon S. Dellink R.. (2015). Integrated medical–psychiatric outpatient care in functional gastrointestinal disorders improves outcome: a pilot study. Eur. J. Gastroenterol. Hepatol. 27, 721–727. doi: 10.1097/MEG.0000000000000335, PMID: 25831133

[ref36] Labus J. S. Bolus R. Chang L. Wiklund I. Naesdal J. Mayer E. A.. (2004). The visceral sensitivity index: development and validation of a gastrointestinal symptom-specific anxiety scale. Aliment. Pharmacol. Ther. 20, 89–97. doi: 10.1111/j.1365-2036.2004.02007.x, PMID: 15225175

[ref37] Lacy B. E. Tack J. Gyawali C. P. (2022). AGA clinical practice update on management of medically refractory gastroparesis: expert review. Clin. Gastroenterol. Hepatol. 20, 491–500. doi: 10.1016/j.cgh.2021.10.038, PMID: 34757197

[ref38] Law M. Bartlett E. Sebaratnam G. Pickering I. Simpson K. Keane C.. (2023). One more tool in the tool belt: a qualitative interview study investigating patient and clinician opinions on the integration of psychometrics into routine testing for disorders of gut-brain interaction. med Rxiv. doi: 10.1101/2023.06.06.23291063 (preprint). PMC11408741 39295648

[ref39] Levy R. L. Olden K. W. Naliboff B. D. Bradley L. A. Francisconi C. Drossman D. A.. (2006). Psychosocial aspects of the functional gastrointestinal disorders. Gastroenterology 130, 1447–1458. doi: 10.1053/j.gastro.2005.11.057 16678558

[ref40] Lovibond S. H. Lovibond P. F. (1995). Manual for the depression anxiety stress scales, vol. 2nd. Sydney, N.S.W. Australia: Psychology Foundation.

[ref41] Luo Y. Keefer L. (2021). Role of psychological questionnaires in clinical practice and research within functional gastrointestinal disorders. Neurogastroenterol. Motil. 33:e14297. doi: 10.1111/nmo.14297, PMID: 34786802

[ref42] Nunnally J. Bernstein I. H. (1994). Psychometric theory. 3rd Edn. New York City: McGraw-Hill.

[ref43] O’Grady G. Varghese C. Schamberg G. Calder S. Du P. Xu W.. (2023). Principles and clinical methods of body surface gastric mapping: technical review. Neurogastroenterol. Motil. 35:e14556. doi: 10.1111/nmo.14556, PMID: 36989183 PMC10524901

[ref44] Palsson O. S. Whitehead W. E. (2013). Psychological treatments in functional gastrointestinal disorders: a primer for the gastroenterologist. Clin. Gastroenterol. Hepatol. 11, 208–216. doi: 10.1016/j.cgh.2012.10.031, PMID: 23103907 PMC3591464

[ref45] Parker G. Brotchie H. (2010). Gender differences in depression. Int. Rev. Psychiatry 22, 429–436. doi: 10.3109/09540261.2010.492391 21047157

[ref46] Pasricha P. J. Grover M. Yates K. P. Abell T. L. Bernard C. E. Koch K. L.. (2021). Functional dyspepsia and gastroparesis in tertiary care are interchangeable syndromes with common clinical and pathologic features. Gastroenterology 160, 2006–2017. doi: 10.1053/j.gastro.2021.01.230, PMID: 33548234 PMC8547190

[ref47] Patel J. S. Oh Y. Rand K. L. Wu W. Cyders M. A. Kroenke K.. (2019). Measurement invariance of the patient health questionnaire-9 (PHQ-9) depression screener in U.S. adults across sex, race/ethnicity, and education level: NHANES 2005-2016. Depress. Anxiety 36, 813–823. doi: 10.1002/da.22940, PMID: 31356710 PMC6736700

[ref48] Raubinger S. Allworth S. Carey S. (2022). When you are living and dying at the same time: a qualitative exploration of living with gastrointestinal motility disorders. J. Hum. Nutr. Diet. 36, 622–631. doi: 10.1111/jhn.13114, PMID: 36420640

[ref49] Riehl M. E. Kinnucan J. A. Chey W. D. Stidham R. W. (2019). Nuances of the psychogastroenterology patient: a predictive model for gastrointestinal quality of life improvement. Neurogastroenterol. Motil. 31:e13663. doi: 10.1111/nmo.13663, PMID: 31206935

[ref50] Rodrigues D. M. Motomura D. I. Tripp D. A. Beyak M. J. (2021). Are psychological interventions effective in treating functional dyspepsia? A systematic review and meta-analysis. J. Gastroenterol. Hepatol. 36, 2047–2057. doi: 10.1111/jgh.15566, PMID: 34105186

[ref51] Rönkkö M. Cho E. (2022). An updated guideline for assessing discriminant validity. Organ. Res. Methods 25, 6–14. doi: 10.1177/1094428120968614

[ref52] Schmulson M. J. Drossman D. A. (2017). What is new in Rome IV. J. Neurogastroenterol. Motil. 23, 151–163. doi: 10.5056/jnm16214, PMID: 28274109 PMC5383110

[ref53] Sebaratnam G. Law M. Broadbent E. Gharibans A. A. Andrews C. N. Daker C.. (2023). It’s a helluva journey: a qualitative study of patient and clinician experiences of nausea and vomiting syndromes. Front. Psychol. 14:1232871. doi: 10.3389/fpsyg.2023.1232871, PMID: 37637892 PMC10457000

[ref54] Shoji T. Endo Y. Fukudo S. (2018). “Psycho-gastroenterology” in Functional dyspepsia: Evidences in pathophysiology and treatment. eds. Tominaga K. Kusunoki H. (Singapore: Springer Singapore), 105–115.

[ref55] Singh P. Ballou S. Rangan V. Katon J. Hassan R. Iturrino J.. (2022). Clinical and psychological factors predict outcome in patients with functional dyspepsia: a prospective study. Clin. Gastroenterol. Hepatol. 20, 1251–1258.e1. doi: 10.1016/j.cgh.2021.07.043, PMID: 34339874

[ref56] Sperber A. D. Bangdiwala S. I. Drossman D. A. Ghoshal U. C. Simren M. Tack J.. (2021). Worldwide prevalence and burden of functional gastrointestinal disorders, results of Rome foundation global study. Gastroenterology 160, 99–114.e3. doi: 10.1053/j.gastro.2020.04.014, PMID: 32294476

[ref57] Spitzer R. L. Kroenke K. Williams J. B. W. Lowe B. (2006). A brief measure for assessing generalized anxiety disorder: the GAD-7. Arch. Intern. Med. 166, 1092–1097. doi: 10.1001/archinte.166.10.1092 16717171

[ref58] Sriken J. Johnsen S. T. Smith H. Sherman M. F. Erford B. T. (2022). Testing the factorial validity and measurement invariance of college student scores on the generalized anxiety disorder (GAD-7) scale across gender and race. Meas. Eval. Couns. Dev. 55, 1–16. doi: 10.1080/07481756.2021.1902239

[ref59] Stanghellini V. Chan F. K. L. Hasler W. L. Malagelada J. R. Suzuki H. Tack J.. (2016). Gastroduodenal disorders. Gastroenterology 150, 1380–1392. doi: 10.1053/j.gastro.2016.02.011 27147122

[ref60] Terwee C. B. Bot S. D. M. de Boer M. R. van der Windt D. A. W. M. Knol D. L. Dekker J.. (2007). Quality criteria were proposed for measurement properties of health status questionnaires. J. Clin. Epidemiol. 60, 34–42. doi: 10.1016/j.jclinepi.2006.03.012 17161752

[ref61] The Rome Foundation. (2016). Appendix A: Rome IV diagnostic criteria for FGIDs. Available at: https://theromefoundation.org/rome-iv/rome-iv-criteria/

[ref62] Tse A. W. Y. Lai L. H. Lee C. C. Tsoi K. K. F. Wong V. W. S. Chan Y.. (2010). Validation of self-administrated questionnaire for psychiatric disorders in patients with functional dyspepsia. J. Neurogastroenterol. Motil. 16, 52–60. doi: 10.5056/jnm.2010.16.1.52, PMID: 20535327 PMC2879830

[ref63] U.S. Department of Health and Human Services Food and Drug Administration. Guidance for industry patient-reported outcome measures: use in medical product development to support labeling claims. (2009). Available at: https://www.fda.gov/regulatory-information/search-fda-guidance-documents/patient-reported-outcome-measures-use-medical-product-development-support-labeling-claims 10.1186/1477-7525-4-79 PMC1629006 17034633

[ref64] Van Oudenhove L. Crowell M. D. Drossman D. A. Halpert A. D. Keefer L. Lackner J. M.. (2016). Biopsychosocial aspects of functional gastrointestinal disorders. Gastroenterology 150, 1355–1367.e2. doi: 10.1053/j.gastro.2016.02.027, PMID: 27144624 PMC8809487

[ref65] van Tilburg M. A. L. Drossman D. A. Knowles S. R. (2021). Psychogastroenterology: the brain-gut axis and its psychological applications. J. Psychosom. Res. 152:110684. doi: 10.1016/j.jpsychores.2021.110684, PMID: 34847487

[ref66] Weijters B. Baumgartner H. Schillewaert N. (2013). Reversed item bias: an integrative model. Psychol. Methods 18, 320–334. doi: 10.1037/a0032121, PMID: 23646990

[ref67] Weltens N. Iven J. Van Oudenhove L. Kano M. (2018). The gut-brain axis in health neuroscience: implications for functional gastrointestinal disorders and appetite regulation. Ann. N. Y. Acad. Sci. 1428, 129–150. doi: 10.1111/nyas.13969, PMID: 30255954

[ref68] Woodhouse S. Hebbard G. Knowles S. R. (2017). Psychological controversies in gastroparesis: a systematic review. World J. Gastroenterol. 23, 1298–1309. doi: 10.3748/wjg.v23.i7.1298, PMID: 28275310 PMC5323455

[ref69] Wouters M. M. Boeckxstaens G. E. (2016). Is there a causal link between psychological disorders and functional gastrointestinal disorders? Expert Rev. Gastroenterol. Hepatol. 10, 5–8. doi: 10.1586/17474124.2016.1109446, PMID: 26569404

[ref70] Wu J. C. Y. (2011). Community-based study on psychological comorbidity in functional gastrointestinal disorder. J. Gastroenterol. Hepatol. 26, 23–26. doi: 10.1111/j.1440-1746.2011.06642.x 21443703

[ref71] Wu J. C. (2012). Psychological co-morbidity in functional gastrointestinal disorders: epidemiology, mechanisms and management. J. Neurogastroenterol. Motil. 18, 13–18. doi: 10.5056/jnm.2012.18.1.13, PMID: 22323984 PMC3271249

[ref72] Wu M.-J. Zhao K. Fils-Aime F. (2022). Response rates of online surveys in published research: a meta-analysis. Comput. Hum. Behav. 7:100206. doi: 10.1016/j.chbr.2022.100206

[ref73] Yusoff M. S. B. Arifin W. N. Hadie S. N. H. (2021). ABC of questionnaire development and validation for survey research. Educ. Med. J. 13, 97–108. doi: 10.21315/eimj2021.13.1.10

