# Supplementary material for: Development and validation of the Alimetry Gut-Brain Wellbeing Survey: a novel patient-reported mental health scale for patients with chronic gastroduodenal symptoms
Source: Front Psychol. 2024 Jul 8;15:1389671. doi: 10.3389/fpsyg.2024.1389671 (PMC11262055; doi:10.3389/fpsyg.2024.1389671)
Supplement: Supplementary file 2 [file Data_Sheet_2.DOCX]

**Supplementary Methods**

**Phase 1. Concept Selection**

The precursory interview study[[1]](https://paperpile.com/c/tLO65L/1mnn1) identified depression, stress, and anxiety as the most important mental health domains to assess clinically in the target patient group. Both patients and clinicians desired a brief scale that combined these three domains. Based on this feedback, validated concepts from existing and widely used depression, stress, and anxiety questionnaires were used to identify the most important concepts for patients with chronic gastroduodenal symptoms. Using existing questionnaires as the basis for concept identification ensured content validity in the new questionnaire.

Design

Concept selection involved a combination of expert feedback and the analysis of psychometric data gathered from a sample of patients with chronic gastroduodenal symptoms, which was collected as part of another multi-national consortium database study, on behalf of the BSGM working group (<https://www.bsmconsortium.com/>). Data collection was conducted in Auckland (New Zealand), Calgary (Canada), and Western Sydney (Australia). Ethics were obtained for each data collection site via: The Auckland Health Research Ethics Committee (AHREC; AH1130), The University of Calgary Conjoint Health Research Ethics Board (REB19-1925), and the Human Research Ethics Committee at Western Sydney (H13541). All participants provided written informed consent.

Sample

The sample consisted of 79 patients (73% female; mean age= 38.1 years, age range= 15-81 years) and 50 healthy controls (62% female; mean age= 39.2 years, age range= 19-84 years). Patients were defined as meeting the Rome IV criteria[[2]](https://paperpile.com/c/tLO65L/i4iey) for functional dyspepsia and/or chronic nausea and vomiting syndrome, whilst healthy controls were defined as having no chronic gastroduodenal symptoms.

Procedure

Participants completed a battery of psychometric questionnaires while participating in another study, using methods described elsewhere[[3]](https://paperpile.com/c/tLO65L/gm6SN). The Patient Health Questionnaire 9 (PHQ-9)[[4]](https://paperpile.com/c/tLO65L/abqiu) was used to measure levels of depression, the Perceived Stress Scale 4 (PSS-4)[[5]](https://paperpile.com/c/tLO65L/VQADD) was used to measure levels of stress, and the Generalised Anxiety Disorder 7 (GAD-7)[[6]](https://paperpile.com/c/tLO65L/bay5I) was used to measure levels of anxiety. These questionnaires were included as they are some of the most widely used and well-validated existing mental health assessment tools and are frequently used in patients with chronic gastroduodenal symptoms[[7,8]](https://paperpile.com/c/tLO65L/SdXoY+QBKvF).

Statistical Analysis

The key concepts from each questionnaire were selected by statistically evaluating the weighting of the concept as a total predictive value to the original scales’ total score, using the following methodology in Python v3.7.

Firstly, the sample of 79 patients was separated into two subsamples: a 75% training sample and a 25% validation sample. Using bootstrapping 1000 random samples with replacement were generated from the original 75% cohort. The 1000 samples were then passed through a univariate feature selection to rank the importance of each item. Iteratively working from one feature to all features, the combination of features was used to train a regression model and then the predicted scores were compared to the actual scores. Results were compiled such that 1000 predictive scores were amassed for each feature value, along with the mean and 95% confidence interval. The subscale of each scale was then determined by allowing in the number of features required to have a mean accuracy of 90%. In the event of edge cases being close to the threshold, feature weighting was compared statistically (using a two-tailed t-test) to ensure each scale weighting was statistically different (no correction for multiple comparisons was performed). The defined subset of items was then trained on the original 75% sample and validated against the testing 25% sample. A valid score was reported if the R^2^ value was greater than 0.9.

An additional confirmatory analysis was conducted to confirm the specificity of the extracted questions to patients compared to healthy controls. This analysis involved repeating the procedure described above in the healthy control group (n= 50). The rankings of the questions were compared between the two groups using two-tailed t-tests.

**Supplementary Results**

**Phase 1. Concept Selection**

During the evaluation of the PHQ-9, results showed five potential factors (items 1, 2, 4, 7, 8) were required to meet the desired threshold of 0.9. However, there was only a marginal difference in accuracy between including either four or five of these items, with accuracy values shifting from 0.89 to 0.9. Upon applying statistical criteria, a t-test was conducted comparing the weighting scores of these items, with a significant difference revealed. Coupled with expert recommendations suggesting the exclusion of questions related to physical symptomatology for this patient population, item 5 (poor appetite/overeating) was ultimately excluded from the scale.

The results from the confirmatory analysis with healthy controls indicated that all scale values were significantly different, providing evidence to support the assertion that this scale is unique and contextualised to patients with chronic gastroduodenal symptoms. This selection of these items increased the specificity and contextualisation of the questions for use in patients with chronic gastroduodenal symptoms, while also allowing for a reduction in clinician and patient burden by including a smaller subset of questions than would typically be completed in separate anxiety, stress, and depression questionnaires.

**References**

[1] [Law M, Bartlett E, Sebaratnam G, Pickering I, Simpson K, Keane C, et al. “One more tool in the tool belt”: A qualitative interview study investigating patient and clinician opinions on the integration of psychometrics into routine testing for disorders of gut-brain interaction. medRxiv 2023. https://doi.org/](http://paperpile.com/b/tLO65L/1mnn1)[10.1101/2023.06.06.23291063](http://dx.doi.org/10.1101/2023.06.06.23291063)[.](http://paperpile.com/b/tLO65L/1mnn1)

[2] [The Rome Foundation. Appendix A: Rome IV Diagnostic Criteria for FGIDs 2016, January 16.](http://paperpile.com/b/tLO65L/i4iey) <https://theromefoundation.org/rome-iv/rome-iv-criteria/>[.](http://paperpile.com/b/tLO65L/i4iey)

[3] [Gharibans AA, Calder S, Varghese C, Waite S, Schamberg G, Daker C, et al. Gastric dysfunction in patients with chronic nausea and vomiting syndromes defined by a noninvasive gastric mapping device. Sci Transl Med 2022;14:eabq3544. https://doi.org/](http://paperpile.com/b/tLO65L/gm6SN)[10.1126/scitranslmed.abq3544](http://dx.doi.org/10.1126/scitranslmed.abq3544)[.](http://paperpile.com/b/tLO65L/gm6SN)

[4] [Kroenke K, Spitzer RL, Williams JB. The PHQ-9: Validity of a brief depression severity measure. J Gen Intern Med 2001;16:606–13. https://doi.org/](http://paperpile.com/b/tLO65L/abqiu)[10.1046/j.1525-1497.2001.016009606.x](http://dx.doi.org/10.1046/j.1525-1497.2001.016009606.x)[.](http://paperpile.com/b/tLO65L/abqiu)

[5] [Cohen S, Kamarck T, Mermelstein R. A global measure of perceived stress. J Health Soc Behav 1983;24:385–96. https://doi.org/](http://paperpile.com/b/tLO65L/VQADD)[10.2307/2136404](http://dx.doi.org/10.2307/2136404)[.](http://paperpile.com/b/tLO65L/VQADD)

[6] [Spitzer RL, Kroenke K, Williams JBW, Lowe B. A brief measure for assessing generalized anxiety disorder: The GAD-7. Arch Intern Med 2006;166:1092–7. https://doi.org/](http://paperpile.com/b/tLO65L/bay5I)[10.1001/archinte.166.10.1092](http://dx.doi.org/10.1001/archinte.166.10.1092)[.](http://paperpile.com/b/tLO65L/bay5I)

[7] [Breedvelt JJF, Zamperoni V, South E, Uphoff EP, Gilbody S, Bockting CLH, et al. A systematic review of mental health measurement scales for evaluating the effects of mental health prevention interventions. Eur J Public Health 2020;30:539–45. https://doi.org/](http://paperpile.com/b/tLO65L/SdXoY)[10.1093/eurpub/ckz233](http://dx.doi.org/10.1093/eurpub/ckz233)[.](http://paperpile.com/b/tLO65L/SdXoY)

[8] [Boeckxstaens GE, Drug V, Dumitrascu D, Farmer AD, Hammer J, Hausken T, et al. Phenotyping of subjects for large scale studies on patients with IBS. Neurogastroenterol Motil 2016;28:1134–47. https://doi.org/](http://paperpile.com/b/tLO65L/QBKvF)[10.1111/nmo.12886](http://dx.doi.org/10.1111/nmo.12886)[.](http://paperpile.com/b/tLO65L/QBKvF)
